# Gut Microbiota Composition Positively Correlates with Sports Performance in Competitive Non-Professional Female and Male Runners

**DOI:** 10.3390/life14111397

**Published:** 2024-10-30

**Authors:** Guy Shalmon, Rawan Ibrahim, Ifat Israel-Elgali, Meitar Grad, Rani Shlayem, Guy Shapira, Noam Shomron, Ilan Youngster, Mickey Scheinowitz

**Affiliations:** 1 Sylvan Adams Sports Institute, School of Public Health, Tel Aviv University, Tel Aviv-Yafo 6997801, Israel; guyshalmon@yahoo.com; 2 Faculty of Medical and Health Sciences, Tel Aviv University, Tel Aviv-Yafo 6997801, Israel; 3 Department of Biomedical Engineering, Faculty of Engineering, Tel Aviv University, Tel Aviv-Yafo 6997801, Israel; 4 Edmond J. Safra Center for Bioinformatics, Tel Aviv University, Tel Aviv-Yafo 6997801, Israel; 5 Pediatric Infectious Diseases Unit, The Center for Microbiome Research, Shamir Medical Center, Tel Aviv 6997801, Israel

**Keywords:** microbiome, gut microbiota, endurance athletes, runners, males, females

## Abstract

There is still a pressing need for further investigation to bridge the gap in understanding the differences in gut microbiota composition between female runners and their male counterparts. We aimed to determine the gut microbiota composition in competitive non-professional female and male runners and to correlate the gut bacteria to performance. Our study included 40 subjects, of which 22 were runners (13 males and 9 females) and 18 control subjects (9 males and 9 females, representing the general population who perform light physical activity with a weekly running volume of ≤5 km per week). Fecal specimens were collected and analyzed for taxonomic profiling to compare species’ relative abundances between males and females based on the results of 16SrRNA analysis. Bacterial alpha and beta diversity were assessed to determine the differences in microbial composition between runners and controls, and between sexes. Each participant underwent a maximal oxygen consumption test and a time-to-exhaustion test at 85% of the measured VO2max. Blood lactate was collected every 5 min during the tests. Bacterial alpha diversity showed a significant difference (*p* = 0.04) between runners and controls. Taxonomic analysis of gut microbiota composition showed a lower *Enterobacteriaceae* abundance and a higher *Methanosphaera* abundance in runners compared with the control group. Ten different bacteria (*Methanosphaera*, *Mitsuokella*, *Prevotellaceae*, *Megamonas*, *Rothia*, *Oscillospira*, *Bacteroides*, *Odoribacter*, *Blautia massiliensis*, *Butyricicoccus_pullicaecorum*) were positively correlated with exercise (VO2max, lactate blood levels, time to exhaustion, and weekly training volume). We found no significant differences in the gut microbiota composition between male and female runners. Gut microbiota composition positively correlates with sports performance in competitive non-professional female and male runners, and female runners show similar gut microbiome diversity to male runners.

## 1. Introduction

Endurance exercise performance reflects a coordinated response of cardiovascular, pulmonary, and neural functions along with the action of exercising muscles. Exercise induces graded increases in heart rate, arterial pressure, cardiac output, myocardial contractility, and rate and depth of respiration.

Exercise significantly contributes to the gut microbial population. Studies have shown that exercise training independently alters the composition and functional capacity of the gut microbiota [1]. Studies investigating the microbiome’s role in athletic performance showed increased microbial diversity and increases in species or metabolites associated with muscle turnover, recovery, and protein breakdown [2,3].

A study [4] has shown that, in the bowel of marathon runners, there is a more significant amount of *Veillonella atypica* bacterium than inactive counterparts. They isolated the strain from marathon runners, implanted it into the intestines of mice, and found a significant increase in running time compared with control animals.

They hypothesized that *Veillonella atypica* metabolizes the lactate produced by skeletal muscles during exercise. The metabolized lactate is converted into propionate, which acts as a substrate for the working muscles.

As with other areas of research, the majority of microbiome studies are male-dominated studies. Studies examining the gut microbiomes of females are limited, and research comparing the gut microbiomes of females and males in the general population is inconclusive [5]. Nevertheless, preliminary animal and human studies have shown sex differences in the microbiome’s composition, possibly due to differences in estrogen concentrations [5]. For example, in a human study conducted by four centers in France, Germany, Italy, and Sweden, a higher level of the Bacteroides-*Prevotella* group was observed in the males than in females [6]. In another study of Chinese family members, which was conducted using group-specific denaturing gradient gel electrophoresis (DGGE) profiling of *Bacteroides* spp., a higher abundance of Bacteroides *thetaiotaomicron* was identified in the males than in the females [7]. Gastrointestinal physiology has also been gender-specific. Sadik et al. showed that gastric emptying, small-bowel transit, and colonic transit were significantly slower in healthy female subjects than in males [8]. In addition, an apparent bi-directional relationship between estrogen levels and the gut microbiota has been identified [9], an association more pronounced in females than males when examined in animal studies [10]. This gender-based difference highlights the potentially central role of the gastrointestinal tract in runners’ performance and might explain sex-derived differences in other physiological and biological processes. To the best of our knowledge, there is a paucity of studies examining the differences between the microbiome composition of female and male runners under identical experimental settings.

While physical activity has been associated with positive effects on the gut microbiota, like greater diversity and abundance of specific probiotic species, competitive endurance sports have been shown to affect it negatively, such as increased I-FABP serum levels indicating more significant gut integrity loss, intestinal distress, an increase in intestinal permeability, and zonulin [11].

There is still a gap in understanding the difference between the gut microbiota composition of competitive endurance athletes and trainees who engage in regular physical activity; the relationship between specific intestinal bacteria and sports performance needs to be better understood.

This study aimed to determine the gut microbiota composition in competitive non-professional female and male runners in a sex-specific manner. We also aimed to examine whether the gut microbial composition expressed in runners correlated with endurance exercise performance.

## 2. Materials and Methods

### 2.1. Study Design and Participants

For our study, we recruited runners from competitive sports groups, while participants in the control group were recruited from the general population.

Forty subjects participated in the study (22 males and 18 females). They included 22 competitive non-professional runners (13 males and 9 females, with a mean age of 43 ± 6.5 years). As controls, we used 18 subjects (9 males and 9 females, mean age of 41 ± 7.4 years) representing the general population who perform light physical activity with a weekly running volume of ≤5 km per week. The runners were competitive endurance athletes who ran at least 50 km per week.

Each participant completed an online questionnaire describing their weekly training workout volume (days of training per week and total minutes per session), exercise intensity (expressed as % of heart rate maximum), and dietary habits (types of foods, amounts, frequency of eating) to learn about their diet, which can affect the gut microbiota population. To reduce dietary variability, we only included omnivorous participants. Subjects who consumed supplements, like probiotics, prebiotics, multivitamins, antacids (beta-alanine, sodium bicarbonate, others), and antibiotics in the three months before the study were excluded. Each subject received information about the study and signed an informed consent form after approval from the Tel Aviv University Ethics Committee, Israel (approval No. 0003766-1). All informed consent forms signed by the subjects are in the files of the principal researcher at Tel Aviv University, Israel.

### 2.2. Exercise Tests

Each participant performed two exercise stress tests: (1) a maximal exercise stress test to evaluate the athletes’ aerobic fitness level and (2) a sub-maximal exercise stress test to determine ‘time to exhaustion’. For the maximal exercise test, maximal oxygen consumption (VO2max) and exercise performance were evaluated using a running test on a treadmill and gas exchange analyses using a COSMED Quark metabolic cart (COSMED S.r.l., Rome, Italy) [12,13]. Heart rate (HR) was monitored using either a POLAR watch (Polar Electro Oy, Kempele, Finland) or a GARMIN watch (Garmin Ltd., Olathe, KS, USA). We started the test with a running speed equal to 50% of the running economy (subjectively assessed). Running speed was increased every minute until reaching VO2max. For the sub-maximal exercise testing to exhaustion, a week later, each subject performed a sub-maximal exercise test at 85% of the measured VO2max until exhaustion [14]. Capillary blood lactate levels were measured from fingertip samples every 5 min during the exercise test using a Lactate Scout+ hand-held analyzer (EKF Diagnostics GmbH, Barleben, Germany).

### 2.3. Gut Microbiome Analysis

#### 2.3.1. Stool Samples Collection

The subjects received a sterile stomacher^®^ bag (Seward Ltd., Worthing, West Sussex, UK) for sample collection on their first visit to the Institute (when they performed the VO2max test) and were requested to bring the fecal specimen at their subsequent visit a week later. Samples were aliquoted in collection tubes and stored within 4 h of sampling at −80 °C pending analysis. The stool preparation was performed under anaerobic conditions.

#### 2.3.2. DNA Extraction

DNA was extracted from samples using 270 µL GT lysis buffer and 30 µL proteinase K (from MagCore Genomic DNA Tissue Kit, RBC Bioscience, Taipei, Taiwan) along with 200 µL sample in bead beating tubes type C (GeneAid, Taipei, Taiwan). Bead beating was performed for 2 min using a Biospec machine (BioSpec Products, Bartlesville, OK, USA). Samples were then incubated at 60 °C for 2 h and extracted on a MagCore machine (RBC Bioscience, Taipei, Taiwan) using MagCore Genomic DNA Tissue Kit cartridges and protocol.

#### 2.3.3. PCR Protocol

DNA was quantified by nanodrop from each tube, and ~20 ng was used as a template for initial PCR. Amplification was performed using Hot Start Ready Mix (PCR Biosystems Ltd., London, UK) using custom primers covering the V4 region primers from Earth Microbiome Project containing CS1/CS2 adaptors [15] for 25 cycles in a volume of 25 µL.

From each sample, 2 µL of PCR1 amplified sample containing CS1/CS2 adaptors was amplified for ten cycles in 10 µL using the Fluidigm Access Array Barcode library according to the manufacturer’s protocol (2 µL barcode per reaction) [16]. DNA was purified using Pure Beads (Roche Sequencing Solutions, Inc., Wilmington, DE, USA) at a ratio of 0.65× and quantified with qubit using dsDNA high sensitivity assay (DeNovix Inc., Wilmington, DE, USA). DNA size and integrity were quantified by TapeStation using Agilent DNA ScreenTape and reagents (Agilent Technologies Inc., Santa Clara, CA, USA).

#### 2.3.4. Sequencing

Samples were run on a MiSeq (Illumina Inc., San Diego, CA, USA) sequencer with 30% PhiX using MiSeq Reagent Kit v2 500PE (Illumina Inc., San Diego, CA, USA) [17]. Demultiplexing was performed using bcl2fastq (Illumina, Inc., San Diego, CA, USA, version 2.20.0.422) with default parameters, allowing for 0 mismatches. Data were then mapped to PhiX using bowtie2 (Johns Hopkins University, Baltimore, MD, USA, version 2.4.5) to remove PhiX control [18], and unmapped reads were quantified, collected, and examined using FastQC (Babraham Bioinformatics, Babraham Institute, Cambridge, UK, version 0.11.9).

#### 2.3.5. Analysis

Demultiplexed reads were analyzed using the QIIME2 pipeline (version qiime2-2020.8) on 16S rRNA gene sequences from microbial communities. The analysis workflow consisted of quality filtration of the sequence data and operational taxonomic unit (OTU) clustering performed using default parameter settings at 97% sequence similarity with the SILVA database (version V132). The adaptor sequences were removed, and read with a quality score lower than 25 or length < 150 bp were discarded. The maximum number of acceptable ambiguous nucleotides was set to two, and chimeric sequences and singletons were also detected and discarded. Subsequently, alpha diversity analysis (using the estimate_richness function along with Faith’s PD) was performed for different sample groups. Faith’s PD [19] is a popular and highly utilized phylogenetic alpha diversity metric that accounts for the phylogenetic relatedness of the community members, and it has been noted to be more sensitive in distinguishing disease factors in the human digestive system relative to other alpha diversity indices [20]. We employed a non-parametric statistical comparison method based on the assumption that the data are not normally distributed and that equal variance cannot be assumed. Specifically, the Kruskal–Wallis test was used to compare the multiple groups (i.e., male runners, female runners, male controls, and female controls). Additionally, beta diversity analysis (utilizing distance functions for unweighted and weighted UniFrac and Bray–Curtis dissimilarities) was conducted for various sample groups, and a principal coordinate analysis (PCoA) plot was generated to present the beta diversity. For differential abundance analysis of gut microbiome, DESeq2 (version 1.36.0) from the R/bioconductor package (version 3.19) was employed. The laboratory with which we collaborated on bioinformatics [Koren Lab] employs the DESeq2 method for microbiome studies. We maintained the same approach and analysis as in previous works to ensure consistency in our methods. Rarified scaled OTUs were labeled by the lowest assigned taxa level possible and summarized per taxa. Differential abundance was assessed, with significant taxa determined by adjusted *p*-values < 0.05 and |log2FoldChange| ≥ 0.58. For differential analysis of the different activities, sex was included as a blocking factor in the design formula. Boxplots were generated using ggplot2 (version 3.4.4). Correlations between the microbiome and clinical parameters were calculated using the corr.test function from the psych package (version 2.3.9) and visualized with the corrplot package (version 0.92). Comparison of BMI, weekly training volume, and cardiorespiratory measures between the groups was conducted using an independent samples *t*-test in IBM SPSS Statistics (version 29).

## 3. Results

### 3.1. Participants’ Characteristics and Cardiopulmonary Exercise Tests

Each participant underwent exercise tests to evaluate their physical fitness. When comparing the two groups, runners vs. controls, the groups had no differences in body mass index (BMI). Table 1 presents each experimental subject’s characteristics, and Table 2 presents the characteristics of the female and male runners. As expected, the runners’ cardiopulmonary exercise test results were higher than the controls (Table 1). Still, we found no statistically significant differences between female and male runners (Table 2).

### 3.2. Microbiome Results

**Figure 1:**
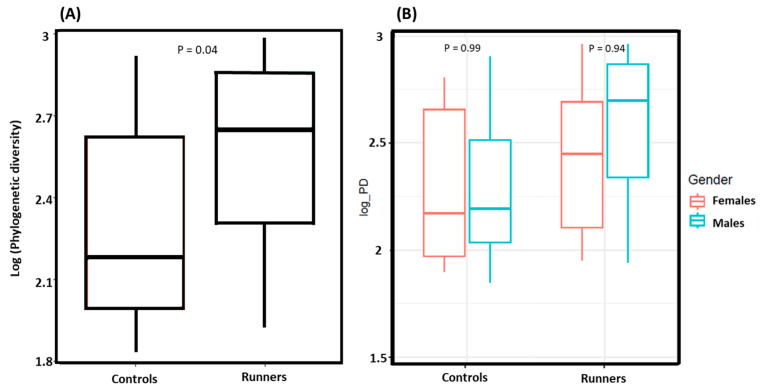
(**A**) Alpha diversity assessment between runners and controls, with runners exhibiting greater species richness than the controls (*p* = 0.04). (**B**) Alpha diversity comparison between male and female runners, male and female controls. No significant differences were observed between males and females within the groups. The alpha diversity assessment utilized the estimate_richness function along with Faith’s PD.

Alpha diversity was significantly higher in runners than controls (*p* = 0.04) (Figure 1). No difference in alpha diversity was observed between males and females in any of the groups.

Beta diversity measures the differences in microbial composition between different samples, providing insights into the variability and distribution of species across various environments. We examined the differences in beta diversity between runners and controls. Our results showed that principal coordinate analysis (PCoA) based on unweighted UniFrac distance revealed differential clustering between the groups, approaching statistical significance (*p* = 0.06). In contrast, analyses using weighted UniFrac distance and Bray–Curtis metrics showed no significant differences (*p* = 0.7 and *p* = 0.4, respectively). Additionally, we found no differences between females and males within each group.

**Figure 2:**
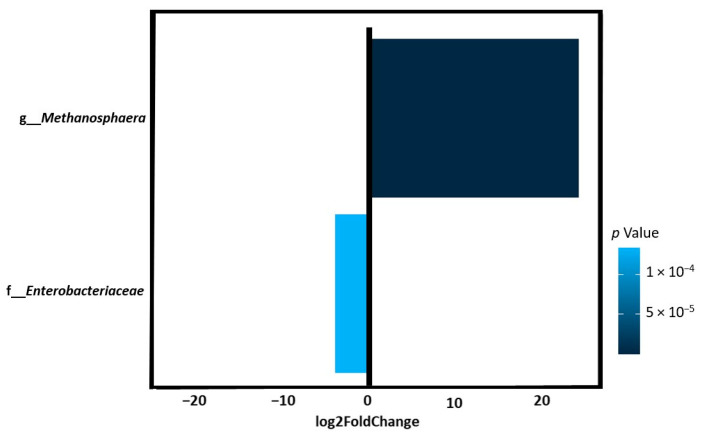
The abundance of selected bacteria in runners compared to controls was identified by DESeq2. Log2FoldChange greater than zero indicated more abundant, whereas log2FoldChange less than zero indicated less abundant. Runners showed a significantly lower abundance of *Enterobacteriaceae* and a significantly higher abundance of *Methanosphaera* than the controls.

Taxonomic analysis of gut microbiota composition revealed a quantitative difference in bacterial types between the study groups. At the family level, runners showed a significantly lower abundance of *Enterobacteriaceae* than control subjects (log2FoldChange = −3.9, *p* = 0.0001). At the genus level, runners showed a significantly higher abundance of *Methanosphaera* (log2FoldChange = 24.01, *p* = 5.64 × 10^−20^) than the controls (Figure 2).

**Figure 3:**
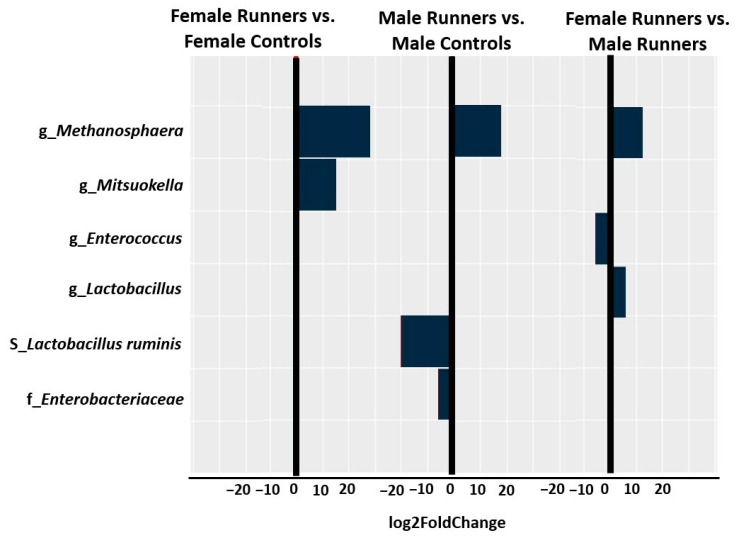
The abundance of bacteria in female and male runners was identified by DESeq2. Log2FoldChange greater than zero indicated more abundant, whereas log2FoldChange less than zero indicated less abundant.

Male runners expressed a significantly lower abundance of *Enterobacteriaceae* (log2FoldChange = −5.7, *p* = 1.27 × 10^−6^) at the family level, a significantly higher abundance of *Methanosphaera* (log2FoldChange = 18.1, *p* = 2.03 × 10^−6^) at the genus level, and a significantly lower abundance of *Lactobacillus_ruminis* (log2FoldChange = −20.3, *p* = 8.99 × 10^−9^) at the species level compared with control group (Figure 3). In contrast, female runners showed a significantly higher abundance of *Methanosphaera* (log2FoldChange = 29.1, *p* = 1.68 × 10^−8^) and a significantly higher abundance of *Mitsuokella* (log2FoldChange = 15.8, *p* = 2.20 × 10^−6^) at the genus level (Figure 3) compared with controls. Compared to male runners, female runners showed a significantly lower abundance of *Enterococcus* (log2FoldChange = −5.8, *p* = 0.0007) but a significantly higher abundance of *Lactobacillus* (log2FoldChange = 6, *p* = 0.0005), and a significantly higher abundance of *Methanosphaera* (log2FoldChange = 11.7, *p* = 2.10 × 10^−9^) (Figure 3).

Microbiome composition is associated with exercise performance: we found a positive correlation between the presence of *Methanosphaera* and lactate blood levels (r = 0.51, *p*-adj = 0.05) and time to exhaustion (r = 0.41, *p*-adj = 0.007). Additionally, we found a positive correlation between the presence of *Mitsuokella* with VO2max (r = 0.41, *p*-adj = 0.05) and time to exhaustion (r = 0.41, *p*-adj = 0.03). Table 3 shows the top 10 positive correlations in runners between bacteria at the species or genus level and physiological measures in exercise. The table includes positive correlations (*p*-adj ≤ 0.05) with a linear relationship to the Pearson correlation coefficient above 0.4 (r > 0.4).

Table 3 shows the correlations between bacteria at the species or genus level and the physiological measures during exercise in runners. The Pearson correlation coefficient (r) quantifies the strength and direction of a linear relationship between two continuous variables, with values ranging from −1 to +1. A weak correlation (0 to 0.3 or −0.3 to 0) indicates minimal association, while a moderate correlation (0.3 to 0.7 or −0.7 to −0.3) suggests a more substantial relationship. Strong correlations (0.7 to 1.0 or −1.0 to −0.7) reflect a robust linear association, where changes in one variable closely relate to changes in the other. Understanding these correlations is essential for interpreting the dynamics between the studied variables.

## 4. Discussion

The current study showed that gut microbiota composition positively correlates with exercise performance in competitive non-professional female and male runners.

Recently, there has been a growing interest in investigating runners’ gut microbiota and its possible association with sports performance. In addition, while regular physical activity is associated with positive effects on the gut microbiota, competitive endurance sport is linked as a factor that negatively affects it, such as increased I-FABP serum levels indicating more significant gut integrity loss, intestinal distress, an increase in intestinal permeability, and zonulin [11]. There is still a gap in understanding the difference between competitive endurance athletes’ gut microbiota composition and recreational physical activity trainees.

Alpha diversity, the major indicator of microbial diversity and species richness in the individual sample [21], was significantly higher in the competitive runners than in the controls. This observation is strengthened by the fact that our control group was not made up of inactive people but individuals who routinely perform moderate amounts of physical activity to promote a healthy lifestyle. However, no differences in alpha diversity were observed in the comparison between male and female runners. This finding may indicate that factors influencing alpha diversity, such as training regimens, diet, or environmental conditions, are consistent across genders in this specific cohort. Furthermore, it may reflect the influence of inherent biological or physiological similarities that minimize divergence in microbial diversity between the two groups.

The taxonomic analysis found a lower abundance of *Enterobacteriaceae* among the competitive runners. The *Enterobacteriaceae* family comprises commensals and opportunistic disease-causing pathogens. However, *Enterobacteriaceae* usually constitute less than 1% of the healthy gut microbiota [22]. Studies have reported an increase in the proportion of potentially harmful *Enterobacteriaceae* family members in IBD patients [23]. Therefore, it would appear that the host’s inflammatory response could trigger gut microbiota imbalance, most likely caused by Enterobacteriaceae blooming, which leads to the persistence of IBD’s inflammatory state [23]. Previous studies support our findings. Lambert et al. [24] showed that in diabetic mice, *Enterobacteriaceae* were less abundant in the exercise group than in diabetic controls following six weeks of exercise or sedentary activity. Munukka et al. [25] showed that six weeks of endurance training had been observed to reduce *Enterobacteriaceae* in overweight women. Therefore, it can be assumed that intensive training may reduce the presence of these potentially pathogenic bacteria.

**Figure 4:**
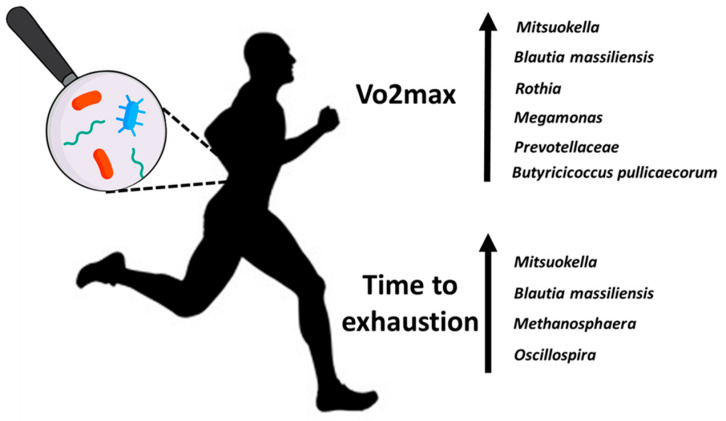
Higher abundances of microbiota depend on the VO2max and time to exhaustion in runners.

We found a higher abundance of eight bacterial populations (Figure 4) depending on the VO2max and time to exhaustion in runners. Interventional studies are needed to examine whether these organisms contribute to this increased exercise performance or are a marker by association.

We identified 10 bacteria that are positively correlated with physiological measures in exercise (Table 3), of which five are butyrate-producers (*Blautia* [26,27], *Prevotellaceae* [28,29], *Oscillospira* [30], *Odoribacter* [31], *Butyricicoccus_pullicaecorum* [32]) and one (*Mitsuokella*) produces lactate, succinate, and acetate. Koike et al. [33] suggested that increasing *Mitsuokella* can elevate lactate production and convert it into butyrate. Butyrate-producing bacteria were also shown in both Allen et al. [34] and Estaki et al. [35] to correlate positively with VO2max and VO2peak, respectively. Butyrate may contribute to athletic performance. The literature suggests that short-chain fatty acids (SCFAs), such as butyrate, could enhance the athlete’s immunity, improve exercise recovery via anti-inflammatory activity, and provide additional energy substrates for exercise performance [36]. Moreover, SCFAs—which modulate the metabolism at various organ sites, including within skeletal muscle—have enhanced endurance exercise capacity in mice [37].

Recent evidence suggests that aerobic exercise improves the diversity and abundance of genera from the Firmicutes phylum, influencing the brain through the ‘gut–brain axis’ [38,39]. Of the ten bacteria that positively correlated with exercise in our study, five of them (*Mitsuokella*, *Megamonas*, *Oscillospira*, *Butyricicoccus pullicaecorum*, and *Blautia massiliensis*) belong to the Firmicutes phylum. This finding highlights Firmicutes’ potential role in mediating exercise’s beneficial effects on gut microbiota composition and the brain.

In our study, the runners showed a higher abundance of *Methanosphaera* than the controls (Figure 2), and we found a positive correlation between *Methanosphaera* with lactate blood levels (r = 0.51, *p*-adj = 0.05) and time to exhaustion (r = 0.41, *p*-adj = 0.007).

The methanogenic Archaea, *Methanosphaera*, has been detected in the human gut microbiota by culture-independent methods [40]. *Methanosphaera* is a spherical-shaped, non-motile archaeon initially isolated from human feces, and it oxidizes hydrogen to reduce methanol and convert it into methane [40]. Methane was shown to slow colonic transit [41]. Triantafyllou et al. [42] reviewed the literature and concluded that the prevalence of methane excretion is greater than previously thought. There is a positive association between breath methane positivity and slower intestinal transit times. Interestingly, Pimentel et al. [43] used a combination of animal studies, explant guinea pig ileum, and clinical data from bowel motility studies of IBS patients to provide strong evidence that methane per se slows intestinal transit time. These findings are interesting since it is known that up to 80% of long-distance runners tend to report gastrointestinal complaints, including upper gastrointestinal disorders (stomach pain, nausea, vomiting) and lower gastrointestinal disorders (abdominal pain or discomfort, bloating, diarrhea) [44,45]. Cardiac output generally increases linearly with exercise intensity. This increase in blood flow can have significant consequences for the digestive system, including ischemia in the gut due to blood flow redistribution [45]. Still, the role of the microbiota in exercise adaptation remains unknown [46]. We hypothesize that there may be a relationship between *Methanosphaera* and the reactions of the digestive system during exercise among runners. Further studies are needed to differentiate between cause and effect.

It is worth noting that alpha diversity is the most commonly used indicator for assessing gut microbiota health [21]. Our research findings indicate that competitive runners have a higher weekly training volume and greater physical fitness [including higher VO2max and longer time to exhaustion] than the controls (Table 1), who perform light physical activity with a weekly running volume of ≤5 km per week. Additionally, we demonstrated (Figure 1) that alpha diversity, an indicator of species richness, was higher in competitive runners than in the controls. Based on these findings, our study suggests that a greater weekly training volume and higher physical fitness may enhance the richness of gut microbiota bacterial strains and contribute to a healthier microbiome and the overall health of the athletes.

One of our research questions was whether the runner’s gut microbiome signature is sex-specific. We hypothesized that, based on gender differences, we would find variations in the gut microbiome between female and male runners. Although female runners showed a lower abundance of *Enterococcus*, a higher abundance of *Lactobacillus*, and a higher abundance of *Methanosphaera* than male runners, this was not enough to drive sex-specific differences in beta diversity (inter-individual microbiome composition). We found no difference in the overall microbiome composition between female and male runners.

Our research sheds light on the gut microbiota composition in competitive non-professional female and male runners and its relationship with sports performance. Generating this knowledge is of interest to scientists, coaches, and runners who work to improve their competition results and reduce recovery time during training. Additional studies are required to examine the contribution of the bacteria we found in our research that correlated with exercise performance.

## 5. Conclusions

In recent years, the gut microbiota of runners and its correlation with sports performance has garnered increasing interest. Our study demonstrates that gut microbiota composition positively correlates with exercise performance in competitive non-professional female and male runners, with female runners exhibiting gut microbiome diversity similar to that of male runners. These findings suggest that gut microbiota may play a crucial role in athletic performance, potentially influencing factors such as energy metabolism, inflammation, and recovery. Furthermore, the gut microbiome signature of runners is likely not sex-specific. Understanding the similarities in gut microbiome composition between genders can provide valuable insights into tailored nutritional and training strategies that optimize performance for all athletes.

## Figures and Tables

**Table 1:** Characteristics of the subjects.

	Runners (*n* = 22)	Controls (*n* = 18)	Overall (N = 40)	*p* Value Runners vs. Controls
Gender				
Females	9 (40.9%)	9 (50.0%)	18 (45%)	
Males	13 (59.1%)	9 (50.0%)	22 (55%)	
BMI				
Mean (SD)	23.2 (2.61)	23.9 (4.01)	23.5 (3.31)	*p* = 0.47
Median (min, max)	22.9 (19.1, 28.7)	22.6 (18.9, 33.0)	22.75 (18.9, 33.0)	
Weekly training volume (km)				
Mean (SD)	67 (15.6)	5 (0)	36 (74.8)	*p* < 0.001
Median (min, max)	60 (50.0, 90.0)	5 (5.0, 5.0)	30 (5.0, 90.0)	
Cardiopulmonary indices				
VT1 (mL/kg/min)	35.86 ± 4.4	26.47 ± 3.9		*p* < 0.001
VT2 (mL/kg/min)	43.2 ± 5.7	31.7 ± 4.5		*p* < 0.001
VO2max (mL/kg/min)	46 ± 6.7	36.7 ± 5.4		*p* < 0.001
TEE (min)	15.43 ± 6.7	7.4 ± 3.1		*p* < 0.001
Lactate max (mmol/L)	8 ± 1.6	7.2 ± 2.8		*p* = 0.2

BMI = body mass index. VT1 = first ventilatory threshold, also known as the aerobic threshold. VT2 = second ventilatory threshold, also known as the respiratory compensation threshold (RCT), and the onset of blood lactate accumulation (OBLA). VO2max = maximal consumption of oxygen. TEE = time to exhaustion. The analysis was performed using an independent samples *t*-test in SPSS.

**Table 2:** Characteristics of the female and male runners.

	Female Runners (*n* = 9)	Male Runners (*n* = 13)
BMI Mean (SD)	21.06 (1.46)	24.61 (2.2)
Weekly training volume (km) Mean (SD)	61.11 (12.6)	71.15 (16.6)
Cardiopulmonary indices		
VT1 (mL/kg/min)	34.25 ± 4.37	36.97 ± 4.38
VT2 (mL/kg/min)	40.52 ± 4.33	45.06 ± 5.93
VO2max (mL/kg/min)	43.87 ± 5.4	47.5 ± 7.4
TEE (min)	15.20 ± 7.83	15.59 ± 6.22
Lactate max (mmol/L)	7.71 ± 1.52	8.33 ± 1.79

BMI = body mass index. VT1 = first ventilatory threshold, also known as the aerobic threshold. VT2 = second ventilatory threshold, also known as the respiratory compensation threshold (RCT), and the onset of blood lactate accumulation (OBLA). VO2max = maximal oxygen consumption. TEE = time to exhaustion. The analysis was performed using an independent samples *t*-test in SPSS. There was no observed statistically significant difference between female and male runners.

**Table 3:** Physiological−bacteria correlations in runners.

Bacteria	Correlated with Weekly Training Volume [km]	Correlated with VO2max [mL/kg/min]	Correlated with Lactate Blood Levels [mmol/L]	Correlated with Time to Exhaustion [min]
*g_Methanosphaera*			r = 0.51	r = 0.41
			*p*-adj = 0.05	*p*-adj = 0.007
*g_Mitsuokella*		r = 0.41		r = 0.41
		*p*-adj = 0.05		*p*-adj = 0.03
*g_Prevotellaceae*	r = 0.41	r = 0.63		
	*p*-adj = 0.05	*p*-adj = 0.01		
*g_Megamonas*	r = 0.43	r = 0.45		
	*p*-adj = 0.04	*p*-adj = 0.03		
*g_Rothia*	r = 0.41	r = 0.65		
	*p*-adj = 0.05	*p*-adj = 0.002		
*g_Oscillospira*				r = 0.43
				*p*-adj = 0.02
*g_Bacteroides*			r = 0.46	
			*p*-adj = 0.02	
*g_Odoribacter*			r = 0.41	
			*p*-adj = 0.05	
*s_Blautia massiliensis*		r = 0.69		r = 0.69
		*p*-adj = 0.006		*p*-adj = 0.005
*s_Butyricicoccus_pullicaecorum*		r = 0.42	r = 0.56	
		*p*-adj = 0.05	*p*-adj = 0.006	

## Data Availability

The data described in this article can be freely and openly accessed at Mendeley Data: https://data.mendeley.com/datasets/yrjp3bg42y/1 (accessed on 15 October 2023).
